# Location-Aware Dynamic Session-Key Management for Grid-Based Wireless Sensor Networks

**DOI:** 10.3390/s100807347

**Published:** 2010-08-04

**Authors:** Chin-Ling Chen, I-Hsien Lin

**Affiliations:** Department of Computer Science and Information Engineering, Chaoyang University of Technology Taichung, Taiwan; E-Mail: s9467601@mail.cyut.edu.tw (I-H.L.)

**Keywords:** attack, grid-based, key management, security, wireless sensor networks

## Abstract

Security is a critical issue for sensor networks used in hostile environments. When wireless sensor nodes in a wireless sensor network are distributed in an insecure hostile environment, the sensor nodes must be protected: a secret key must be used to protect the nodes transmitting messages. If the nodes are not protected and become compromised, many types of attacks against the network may result. Such is the case with existing schemes, which are vulnerable to attacks because they mostly provide a hop-by-hop paradigm, which is insufficient to defend against known attacks. We propose a location-aware dynamic session-key management protocol for grid-based wireless sensor networks. The proposed protocol improves the security of a secret key. The proposed scheme also includes a key that is dynamically updated. This dynamic update can lower the probability of the key being guessed correctly. Thus currently known attacks can be defended. By utilizing the local information, the proposed scheme can also limit the flooding region in order to reduce the energy that is consumed in discovering routing paths.

## 1. Introduction

In recent years, there have been major advances in the development of wireless sensors and IC process technology. Due to these advances, wireless sensor networks (WSNs) have been replacing traditional network technologies [1–4]. These WSNs have a number of advantages over wired networks, such as ease of deployment, extended transmission range, and self-organization.

There are, however, a few inherent limitations to WSNs. These include low communication bandwidth, small storage capacity, limited computation resources, and limited device energy. In terms of energy, many researchers assume that all nodes in a sensor network are battery-driven [5,6]. Because of this, energy is a very scarce resource in sensor networks. Therefore, energy efficiency is an important design issue in WSNs.

**Figure 1.**
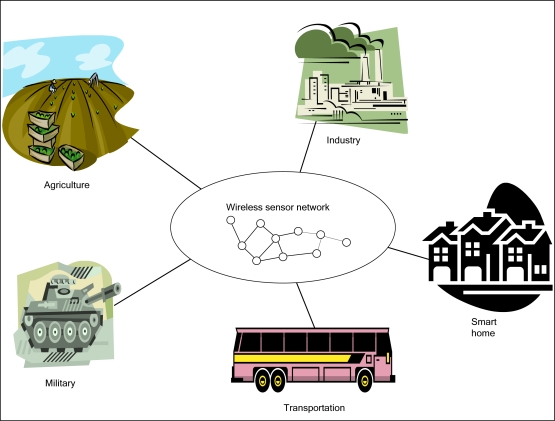
Wireless sensor network applications.

Currently, WSNs are used in various applications. Figure 1 shows a schematic of applications for WSNs. Among their many applications, they can be used in the military, in agriculture, in transportation, in manufacturing, and in smart homes.

Generally speaking, a WSN consists of hundreds or thousands of densely populated sensor nodes that sense the environment they are in and collaboratively work to process and route sensor data. These sensor nodes relay data streams to base stations either periodically or based on events. A base station can be stationary or mobile. If it is mobile, it can move among the sensors and collect data. In a network densely populated with sensor nodes, the area detected by the sensors may overlap, and thus the data sensed by the sensors may be similar. Many wireless collisions occur in this type of network.

The general characteristics of a wireless sensor network include the following: ability for multiple deployments, low cost, small size, and adequate battery power supply. In addition, there are two types of structures for routing transmissions in a wireless sensor network:

1. Cluster: The cluster structure is the most representative of the routing protocols. The general concept behind the cluster structure is to group a large number of sensors into several clusters. In each cluster, a node is chosen as the cluster header. The cluster header collects information from the other sensor nodes within that cluster and transmits the information to the base station.
2. Chaining: The chaining structure differs from the cluster structure in that each detector node in a network is linked together to form a chain. In each round, a node in the chaining structure is chosen as the chain header. Nodes on both ends of the chain transmit data to adjacent nodes in the direction of the chain header, and each receiving node gathers the information. Finally, the chaining header transmits the information to the base station.

**Figure 2.**
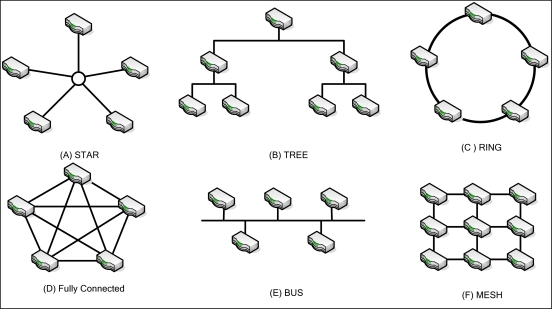
Network topology.

One of several basic network topologies may be used in WSN. The basic network topologies are shown in Figure 2. These include the star, tree, ring, fully connected, bus, and mesh topologies. Different topologies have different features or difficulties. Fully connected networks suffer from problems of NP-complexity. If nodes are added to this topology, the number of links increases exponentially. Mesh networks are regularly distributed networks that generally allow transmission only to a node’s nearest neighbors. In the star topology, all nodes are connected to a single hub node.

It is important to choose the right typology for a network. The type of topology used is important because it affects many network characteristics, such as robustness, energy consumption, and latency. The complexity of data routing and processing also depends on the topology.

Moreover, it is also very important to choose a proper encryption system to protect any transmitted messages. Due to the limited computation resource of a wireless sensor node, public key algorithms (such as Diffie-Hellman key management [7] or the RSA mechanism [8]) are not feasible for solving security problems. We therefore propose a low cost dynamic session-key management for grid-based wireless sensor networks.

## 2. Related Works

In this section, we will review the existing key management protocols for wireless sensor networks. We have classified these protocols into five types: the Random Key Pre-distribution Protocol, the Group-based Key Pre-distribution Protocol, the Hierarchical Key Pre-distribution Protocol, the Direct Communication Protocol, and the Grid-based Routing Protocol.

### 2.1. Five Types of Key Management Protocols

#### (1) Random Key Pre-distribution Protocol

This protocol has three steps: key pre-distribution, key sharing, and key establishment. Before the deployment of any sensor node, m sets of keys are selected from a large key pool. The m keys form a key chain which is sent to each sensor node. One key is selected from among the nodes and is used to transmit data within the group. However, each sensor node must store m keys.

Blom’s method [9] used a global matrix pool to replace the global key pool. In the key pre-distribution phase, each node randomly selects several matrices from the global matrices pool. In this case, any two adjacent nodes have a row of elements from the same matrix that can establish a pair-wise key.

In 2003, Pietro *et al*. [10] proposed a random key transmission protocol. The random keys are transmitted between the sensor nodes such that any two nodes can establish a secure communication channel. The shortcoming of this method is that each sensor node must store more than three sets of keys. In order to enhance security, the number of keys must be increased. However, an increase in the number of keys increases the load of the sensor nodes. Power consumption is also increased.

#### (2) Group-based Key Pre-distribution Protocol

The Group-based Key Pre-distribution Protocol is used to divide the area where the nodes are into several sections. Nodes are placed or dropped into a pre-defined area such that the sensor nodes have a higher probability of good communication.

Liu and Ning [11] proposed a paired key protocol which uses a polynomial key pool and pre-distribution of a grid key. This protocol has higher elasticity in catch and attack. It also has superior sensor node communication. However, the key management algorithm is relatively complicated. More time is required to generate a key.

#### (3) Hierarchical Key Pre-distribution Protocol

The hierarchical key pre-distribution protocol’s elements include a base station, a cluster header, and sensor nodes. Before deployment, each cluster header stores keys. After deployment, the nodes in a network will exchange identification codes. At the same time, that the identification codes are being exchanged, the cluster header will be informed of the identification codes of the sensor nodes. The sensor nodes in the whole network can then communicate with each other. However, if one of the nodes is caught, the information transmitted between the cluster header and the sensor nodes can be easily observed by an enemy. Therefore, the cluster header must increase the number of keys to improve security. The resources of the sensor nodes are limited, however, making an increase in the number of keys impracticable.

Cheng and Agrawal [12] proposed an improved key distribution mechanism (IKDM) which makes use of bivariate polynomials to develop a secure wireless sensor network. In this scheme, each gateway does not directly store the gateway keys of the nodes. Instead, each gateway stores bivariate polynomial functions. After deployment, a node sends its identification code and the gateway numbers to the nearest gateway. Then the gateway receiving the data asks other gateways to obtain sub-keys. The nearest gateway can then compute the gateway keys of its neighboring nodes from these sub-keys. Other similar schemes, such as the one by Jolly *et al*. [13], are also based on the Identity-Based symmetric keying scheme.

#### (4) Direct Communication Protocol

In direct communication in WSNs [14,15], all sensor nodes both gather data and then as well as transmit the collected data directly to the base station. This is extremely energy inefficient, since path loss in wireless systems is proportional to *R^n^*, where *R* is the distance between a sensor node and the base station, and *n* typically ranges between 2 and 4. Therefore, because of energy inefficiency, long distance transmission in direct communication consumes more power. In addition, in a network which consists of a large number of sensor nodes, direct communication may not be feasible because of the large number of collisions. Multiple access schemes can be used to reduce the number of collisions, but the radio bandwidth of each sensor node would be reduced. Alternatively, direct communication can only be used in a small area with just a few sensor nodes in that area.

#### (5) Grid-based Routing Protocol

In recent years, many researchers [15–20] have studied how to use efficient grid-based data dissemination protocols for base stations. In the following section, we will introduce the relevant works.

##### (5.1) Two-Tier Data Dissemination

**Figure 3.**
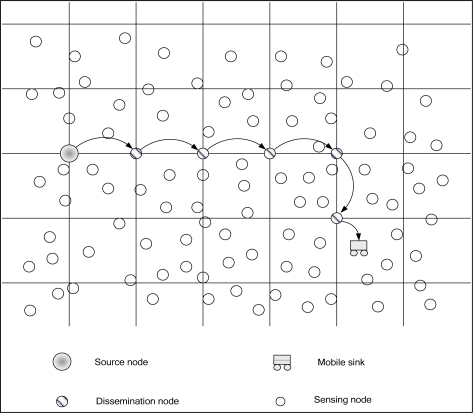
The TTDD scheme for a source node forwarding data to a mobile base station.

A Two-Tier Data Dissemination (TTDD) approach [15,16] provides scalable and efficient data delivery to multiple mobile base stations. The mobile base stations are in constant motion because they are used to build a two-tier structure in sensor networks. TTDD exploits local flooding within a local cell of a grid, which the sources build proactively. Each source uses the nodes on the grid line to transmit data to the base station. However, TTDD does not optimize the path from the source to the base stations. Also, TTDD frequently resumes establishing the entire path to the base stations when the path is down. Figure 3 shows the data forwarding process in TDD.

##### (5.2) Coordination-Based Data Dissemination Protocol for Wireless Sensor Networks

Mobile base stations using flooding to send queries to the nearest grid points. Queries are routed along the grid and data is traced along the path back to the base stations. As a result, the control overhead introduced by base station mobility is limited to the grid cell where a base station is located.

**Figure 4.**
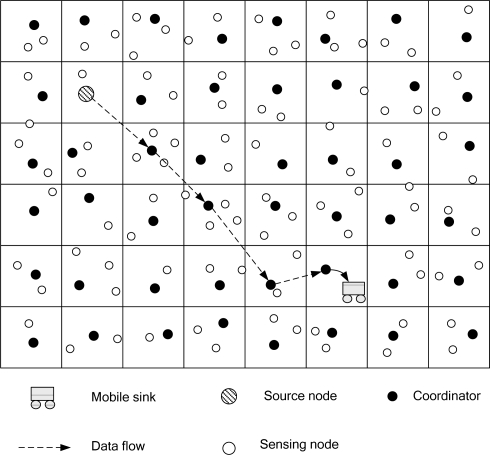
The CODE scheme for multi-hop routing through coordinators.

A coordination-based data dissemination protocol for wireless sensor networks (CODE) [17] considers energy efficiency and network lifetime, especially for sensor networks with high node density. CODE uses a grid structure to establish an efficient data dissemination path between the sources and a mobile base station. Figure 4 shows a grid structure that is used to transmit data. A sensor node is selected to be a coordinator in each grid. When the mobile base station sends a query to the source node, the source node will receive the query and will then transmit data to the mobile base station.

##### (5.3) Data Aggregation for Range Queries

**Figure 5.**
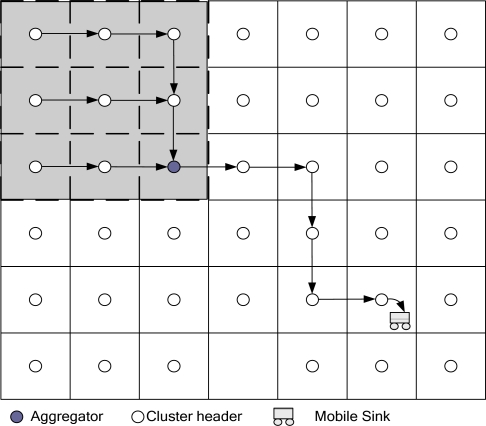
The DARQ scheme for data aggregation with regular-shape ranges.

Chen *et al*. [20] presented an efficient data aggregation algorithm for range queries (DARQ). DARQ is based on a grid structure. Figure 5 shows a DARQ scheme in which sensor nodes can determine their own locations using GPS.

In Chen’s scheme, a mobile base station makes requests for the source to aggregate regular-sharp data. When a source receives a query packet, it constructs an aggregate data tree. This scheme is able to aggregate data in the sensor field with void regions. Void regions are regions in which there are no nodes in a grid because obstacles exist in the grid, no node is deployed, or a node has already died. Chen’s scheme utilizes the proposed face routing scheme [21] to discover where the void regions are and make a detour to avoid the void regions. When a node cannot deliver packets by greedy-forwarding, it uses face routing to make a detour to avoid the void regions.

Our proposed scheme to generate dynamic key management is based on the DARQ scheme, one-way hash function, “two-way” mutual authentication, and symmetric encryption mechanism. A new key will be generated from the previous two keys when a sensor node transmits data in each transaction. The new key will be used for encryption to protect the gathered data. When the sensor node transmits data to a cluster node, the cluster node will request the decryption key of the sensor node from the base station. Since the base station has recorded two primary keys for all sensor nodes, it will transmit the required keys of that sensor node to the cluster node. After receiving the primary keys, the cluster node can decrypt the protected keys.

When the number of sets of the received data is larger than a threshold value *t*, the data will be encrypted and transmitted to the base station. In order to ensure information security, the method for generating the keys for the sensor nodes is the same as the method for the cluster node. In addition, one of the keys transmitted between the base station and the cluster node and another key transmitted between the base station and the sensor nodes will be updated dynamically in order to improve network security.

Although Liao *et al*. [22] solved the flooding problem [15], they did not solve the security problem. This paper investigates the security problem in a grid-based routing protocol by exploiting local flooding within a local cell of a grid which sources build proactively.

### 2.2. Symmetric and Asymmetric Cryptography

Public-key cryptography has been deemed computationally expensive for small sensor nodes, and traditional public-key algorithms (such as RSA) require extensive computation. As a result, public-key cryptography is not considered feasible for small sensors [23]. However, recent progress in Elliptic Curve Cryptography (ECC) [24] provides new opportunities to utilize public-key cryptography in sensor networks. ECC offers security equivalent to that of public-key cryptography using much smaller key sizes. ECC is especially attractive for constrained wireless devices because the smaller keys result in memory, bandwidth and computational savings. NIST [25] has listed the equivalent key sizes for symmetric and asymmetric cryptography, as Table 1 shows:

As we known, the security of ECC rests on the difficulty of the elliptic curve discrete logarithm problem. Recently, Mizanur Rahman and El-Khatib [23] proposed a private key agreement and secure communication for heterogeneous sensor networks which is based on pairing-based cryptography over an elliptic curve. Using this protocol, any two nodes that need to communicate can independently compute the same secret key by using pairing and identity-based encryption properties.

### 2.3. Global Positioning System (GPS)

The Global Positioning System (GPS) is a system which is able to give the exact location of an object on the Earth at anytime, in any weather, and in any location. It is a satellite-based, radio navigation system. The satellite used by the GPS system is continuously monitored by ground stations located worldwide. The satellites transmit signals that can be detected by anyone with a GPS receiver. Using the receiver, one can determine the location of an object with great precision [26–29].

GPS has three parts: a space segment, a user segment, and a control segment. The space segment consists of 24 satellites, each in its own orbit 11,000 nautical miles above the Earth. The user segment consists of receivers which can be hand-held or mounted in a car. The control segment consists of ground stations (five ground stations located around the world) that make sure the satellites are working properly. GPS receivers typically work well outdoors, with positioning accuracy within a 15 meter range.

### 2.4. Basic Assumptions

In this section, we present the basic model for sensor networks. The network model uses the following basic assumptions:

1. After deployment, sensor nodes remain stationary at their initial locations.
2. Each sensor node is assumed to be aware of its own geographic location. Sensors and mobile base stations can determine their own locations using GPS [27–29] (or another method for determining locations [19,20,30–33])
3. Sensor nodes communicate with base stations by delivering data across multiple hops [34]. That is to say, sources and base stations are typically much further apart than a single radio radius.
4. The sensor nodes are homogeneous, and wireless channels are bidirectional. Each sensor node has limited battery energy.
5. The sensor nodes are assumed to know a network’s location which is in the interest region.

## 3. The Location-aware Dynamic Session-key Generation for Grid-based WSNs Scheme

**Figure 6.**
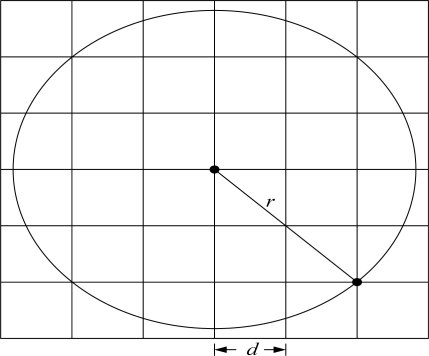
Grid structure.

In this paper, we propose a novel scheme for grid-based generation of a dynamic key to improve the security of previous methods. Our protocol is based on grid-based sensor networks. If a sensor node is selected from the sensors in a grid to announce the selection result and is used for routing, it is called a cluster node. Each base station can obtain information on an event from a grid header. If the base station is interested in the event, it queries the source via the grid header. In the interest region, the base station designates the range for data aggregation. The proposed scheme can defend against various attacks and reduce energy consumption. Figure 6 shows the grid structure.

### 3.1. Eliminating the Broadcast Storm Effect

Broadcast storm effects may occur. To reduce the broadcast storm effect, each node will first broadcast its information to its neighboring nodes. The locations of the source and the interest region (see Section 3.3) will be used to limit the forwarding region. Thus, the broadcast storm problem can be reduced to a certain degree. However, if the forwarding zone is large, there will be a lot of redundancies, contentions, and collisions in the zone. In our protocol, the parameter range in a query packet is used to limit the forwarding zone.

**Figure 7.**
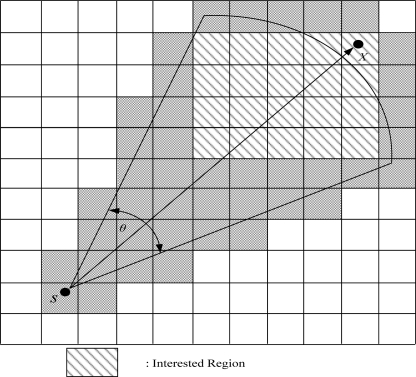
The flooding region.

The forwarding zone can be defined and limited. Let *S* and *X* be the source and destination of the cluster node. The forwarding zone Fan (*θ*, *r*), then, is an area in the shape of a fan from the grid *S* to the grid *X* with angle *θ* and radius *r* [22], as shown in Figure 7.

### 3.2. Grid Formation

**Figure 8.**
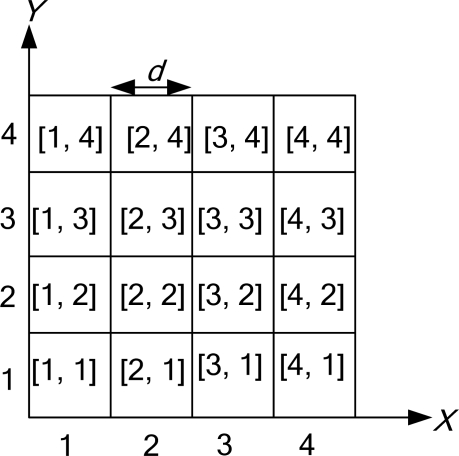
Grid index.

The entire area of a wireless sensor network is partitioned into a 2D logical grid (a 4 × 4 grid, as illustrated in Figure 8). Each grid is a square of size *d* × *d*. Grids are identified (*x*, *y*) using the conventional x y-coordinate system. So that it is aware of its location, each node is equipped with a positioning device, such as a GPS receiver, from which it can read its current location. For any given location, there is a predefined mapping of the location to its grid coordinate. Each grid ID, which is given by [*CX*, *CY*], is assigned as follows: in the first row, from left to right, the grid IDs are [1, 1], [2, 1], [3, 1], and [4, 1]. In the second row, the grid IDs are [1, 2], [2, 2], [3, 2], and [4, 2], and so on. Based on the coordinate (*x, y*), each node computes its *CX* and *CY* as follows:

(1) $$\mathrm{CX}=\left\lfloor \frac{x}{d}\right\rfloor ,\;CY=\left\lfloor \frac{y}{d}\right\rfloor$$

where *d* is the grid size, and *CX* and *CY* are the largest integers not greater than $\frac{x}{d}$and $\frac{y}{d}$, respectively.

Using Equation (1), each node determines which grid it belongs to. Each node will also maintain a neighbor table. The neighbor table is generated using the periodic HELLO protocol [28] at the beginning of a network life. The HELLO packet is small. In addition, the HELLO overhead from the periodic HELLO protocol is very small.

Let *r* be the transmission distance of a radio signal. We use the maximum value $d=r/2\sqrt{2}$. The maximum value *d* of a cluster node is located at a grid and is capable of talking to any of the cluster nodes of its 8 neighboring grids. However, a smaller *d* also means more cluster nodes in the network, which in turn implies a higher overhead for delivering a packet, as well as more broadcast storm. Thus, there exists some tradeoff in choosing a moderate *d* value.

**Figure 9.**
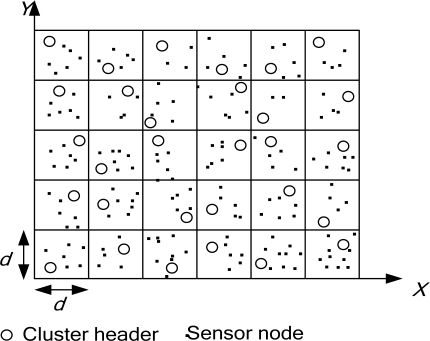
A physical area partitioned into logical grids

In each grid, one sensor node is selected to be the header of that grid. We call a node a “header” when it has more remaining energy than other nodes in that grid. Figure 9 shows a physical area partitioned into logical grids.

### 3.3. Selection of an Interest Region

When an interesting event happens in the selected region, a sensor node will be conscious of this event. After the event, a sensor node will broadcast a packet to find one-hop neighboring nodes. If a neighboring node is conscious of the event, it will forward this packet and store this message in an events table. We describe the event process below.

Step 1: When an event of interest happens in the interest region, a sensor node will be conscious of this event. The sensor node will broadcast a packet to its one-hop neighboring nodes.

Step 2: After receiving the packet, the neighboring nodes will be conscious of the event and will go to the next step; otherwise, the neighboring nodes will drop this packet.

Step 3: The neighboring nodes will forward the packet and store this message in their events table.

**Figure 10.**
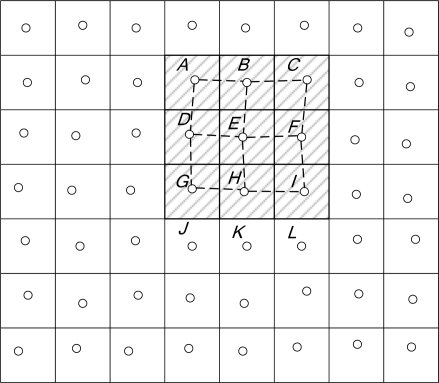
Selected interest region.

For example, in Figure 10, node E is conscious of an event occurring in its region. Node E will broadcast a packet to its one-hop neighboring nodes A, B, C, D, F, G, H and I. Because Nodes A, B, C, D, F, G, H and I are now conscious of the event in his region after receiving the packet, they will forward this packet and store this message in their events table. Nodes J, K, and L will drop this packet since they are not conscious of the event.

### 3.4. Cluster Node Election

To make certain that the cluster node stays alive in each grid, an efficient method for cluster node selection is necessary. Residual energy is used in the selection of the cluster node. In each grid, when the original cluster node residual energy is less than the assumed threshold, one node will be selected as the cluster node for that grid. To maintain the quality of routes, we let the cluster node of a grid be the node with the largest residual energy in that grid.

After a sensor node detects an interest region, the cluster node is selected. The steps for selecting a cluster node are follows:

Step 1: In the interest region, each sensor node sends a cluster node selection request packet < node_id, Grid_id, Residual_Energy, Timeout > to the other nodes, where Grid_id is an identification code for the grid and Residual_Energy is the residual energy of a node in a grid. If the time it takes for a cluster node to receive the selection request packet is greater than the Timeout value, then the packet is discarded.

Step 2: When a node receives the cluster node selection request packet, the node judges whether it has the largest residual energy. If it does, the node becomes a cluster node; otherwise, the request packet is discarded.

The cluster node will be selected periodically to keep the gateway from running out of energy.

The main feature of our scheme is as follows. We assume that a cluster node has the most residual energy. When a cluster node detects an interesting event, it will broadcast a packet to all the cluster nodes. Thus, all the cluster nodes will know whether an event has occurred. When a base station wants to know whether there has been an interesting event, it sends a request packet to ask the cluster node in its grid. When the cluster node receives the request packet, it sends a reply packet to the base station.

### 3.5. Notation

The notations used in our scheme and in this paper are given below, along with their meaning.

*h( )*: the one-way hash function, used for key generation.
*a_i_, a_i−1_*: two parameters used for generating a key which is pre-deployed in the ith sensor node, and *a_i+1_* *= h (a_i_)*.
*b_i_, b_i−1_*: two parameters used for generating a key which is pre-deployed in the ith cluster node.
*N_1_, N_2_, N_3_*: three nonces.
*K_si_*: the ith key of the sensor node.
*K_ci_*: the ith key of the cluster node.
*K_msg_*: the key used for encrypting or decrypting the updated-key message msgfinish.
*Seed*: the seed for updating the key which is pre-deployed in each of the sensor nodes.
*ID_si_*: the identity of the ith sensor node.
*ID_ci_*: the identity of the ith cluster node.
*ID_Bi_*: the identity of the ith base station.
*C_si_*: the encrypted information generated by the ith sensor node.
*C_ci_*: the encrypted information generated by the ith cluster node.
*C_b_*: the encrypted information generated by the base station.
*ID_list_*: the identity set list of the t sensor nodes received from the cluster nodes, such as *ID_list_* = (*ID_s1_*, *ID_s2_*,…,*ID_st_*)
*K_list_*: the key of the sensor nodes generated by the cluster node, such as *K_list_* = (*K_s1_*, *K_s2_*,…,*K_st_*)
*M_i_*: the plaintext information is generated by the ith sensor node.
*M_f_*: the latest information received by the base station.
*E(M,K)*: the symmetric encryption infrastructure makes use of key *K* (for example, AES-128 bits) to encrypt *M*.
*D(M,K)*: the symmetric decryption infrastructure makes use of key *K* (for example, AES-128 bits) to decrypt *M*.
*A? = B*: determine whether A equal to B.

### 3.6. Communication Protocol

**Figure 11.**
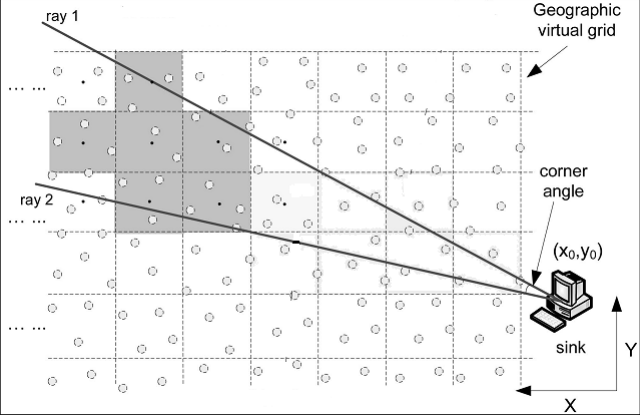
Transmission paths for the sensor network.

In our proposed protocol, we use a dynamic key management mechanism. In this mechanism, two keys are preset in each sensor node. These two keys generate a new key for the next round, and will also be preset in the cluster node. The generation of the session key will be the same as the generation of the key in the sensor node. Using this key management mechanism, we can thus ensure the security of the data transmission. The transmission paths of the sensor network are shown in Figure 11.

**Figure 12.**
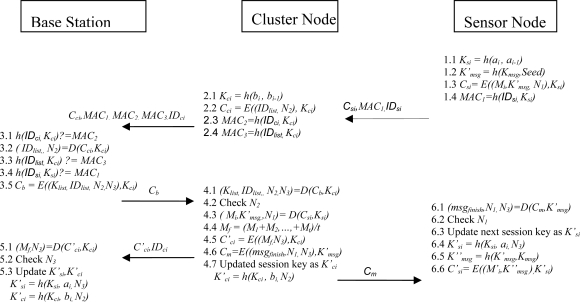
The communication protocol.

We divide our protocol into the following steps, as shown in Figure 12.

Step 1: When the deployed sensor node *i* returns the collected data *M_i_*, the sensor node will make use of the preset parameters *a_i_* and *a*_*i*−1_ to generate a key, *K_si_*, where

(2) $$K_{\mathrm{si}}=h(a_{i},a_{i-1})$$

Furthermore, the two parameters *K_msg_* and the Seed preset in each of the nodes will use the hash function to generate a new message key, *K′_msg_*, where:

(3) $$K_{\mathrm{msg}}^{\prime }=h(K_{\mathrm{msg}},\mathrm{Seed})$$

At that time, the sensor node generates *N*_1_ and makes use of *K_si_* to encrypt the collected data *M_i_*, the preset *K′_msg_*, and *N*_1_ into packet *C_si_* as follows:

(4) $$C_{\mathrm{si}}=E((M_{i},K_{\mathrm{msg}}^{\prime },N_{1}),K_{\mathrm{si}})$$

The sensor node also computes the message authentication code *MAC_1_* as follows:

(5) $${\mathrm{MAC}}_{1}=h({\mathrm{ID}}_{\mathrm{si},}K_{\mathrm{si}})$$

The (*C_si_*, *MAC*_1_, *ID_si_*) is then transmitted to the cluster node.

Step 2: When the cluster node receives more than *t* packets, or when the period is longer than a specified time, the cluster node will record and transmit the identity, *ID_si_*, of the sensor node. It will also arrange a list, *ID_list_*

(6) $${\mathrm{ID}}_{\mathrm{list}}=({\mathrm{ID}}_{s1},{\mathrm{ID}}_{s2},\ldots ,{\mathrm{ID}}_{\mathrm{st}})$$

The cluster node will make use of the two preset parameters *a_i_* and *a*_*i*−1_ to generate a key, *K_ci_*, where

(7) $$K_{\mathrm{ci}}=h(a_{i},a_{i-1})$$

At that time, the cluster node will generate *N*_2_ and make use of *K_ci_* to encrypt *ID_list_* and *N*_2_

(8) $$C_{\mathrm{ci}}=E(({\mathrm{ID}}_{\mathrm{list}},N_{2}),K_{\mathrm{ci}})$$

After that, the cluster node computes the following message authentication code *MAC_2_* and *MAC_3_* as follows:

(9) $${\mathrm{MAC}}_{2}=h\;({\mathrm{ID}}_{\mathrm{ci},}\;K_{\mathrm{ci}})$$

(10) $${\mathrm{MAC}}_{3}=h\;({\mathrm{ID}}_{\mathrm{list},}\;K_{\mathrm{ci}})$$

The cluster node sends (*C_ci_*, *MAC_1_*, *MAC_2_*, *MAC_3_*, *ID_ci_*) to the base station.

Step 3: After receiving the packet from the cluster node, the base station will seek the corresponding key *K_ci_* to verify the cluster node’s identity and decrypt *C_ci_* as follows:

(11) $$h\;({\mathrm{ID}}_{\mathrm{ci},}\;K_{\mathrm{ci}})?={\mathrm{MAC}}_{2}$$

(12) $$\left({\mathrm{ID}}_{\mathrm{list}},N_{2})=D(C_{\mathrm{ci}},K_{\mathrm{ci}}\right)$$

Next, the base station will check the integrity of the *ID_list_* using

(13) $$h\;({\mathrm{ID}}_{\mathrm{list},}\;K_{\mathrm{ci}})?={\mathrm{MAC}}_{3}$$

Based on the *ID_list_*, the base station will search for the corresponding key *K_si_* and arrange them into the key list *K_list_*, where *K_list_*= (*K*_*s*1_, *K*_*s*2_,…, *K_st_*). It will verify the sensor node’s identity using

(14) $$h\;({\mathrm{ID}}_{\mathrm{si},}\;K_{\mathrm{si}})?={\mathrm{MAC}}_{1}$$

If the above verifications fail, this packet will be discarded.

At that time, the base station will generate *N*_3_ and make use of *K_ci_* to encrypt(*K_list_*, *ID_list_*, *N_2_*, *N_3_*) The encrypted data *C_b_* will be sent to the cluster node, where

(15) $$C_{b}=E((K_{\mathrm{list}},{\mathrm{ID}}_{\mathrm{list}},N_{2},N_{3}),K_{\mathrm{ci}})$$

Step 4: When the cluster node receives the response data *C_b_* from the base station, it will make use of the key *K_ci_*, which is generated by itself, to decrypt *C_b_*

(16) $$\left(K_{\mathrm{list}},{\mathrm{ID}}_{\mathrm{list}},N_{2},N_{3})=D(C_{b},K_{\mathrm{ci}}\right)$$

The cluster node will then check whether *N*_2_ is equal to the *N*_2_ generated in step 2.

The cluster node can only use the ith key *K_si_* of the sensor node of *K_list_* to decrypt *C_si_*; otherwise, this packet is discarded.

(17) $$\left(M_{i},K_{\mathrm{msg}}^{\prime },N_{1})=D(C_{\mathrm{si}},K_{\mathrm{si}}\right)$$

After that, the cluster node will calculate the average value of each set of data and obtain *M_f_* as follows:

(18) $$M_{f}=(M_{1}+M_{2}\;+,\ldots ,+\;M_{t})/t$$

This ensures that the data is accurate when it is transmitted to the backend. This cluster node will make use of *K_ci_* to encrypt *M_f_* and *N*_3_ into *C′_ci_*,

(19) $$C_{\mathrm{ci}}^{\prime }=E((M_{f},N_{3}),K_{\mathrm{ci}})$$

The cluster node identity *ID_ci_* along with *C′_ci_* are transmitted together to the base station. At that time, the cluster node will update the session key into *K′_ci_* for the next round.

(20) $$K_{\mathrm{ci}}^{\prime }=h(K_{\mathrm{ci}},a_{i})$$

Furthermore, the cluster node will make use of the key *K′_msg_*, transmitted from the sensor node, to encrypt the transmitted update message *msg_finish_* as follows:

(21) $$C_{m}=E(({\mathrm{msg}}_{\mathrm{finish}},N_{1},N_{3}),K_{\mathrm{msg}}^{\prime })$$

The encrypted packet *C_m_* will then be broadcasted to the sensor nodes to inform the sensor nodes that message transmission is completed. The cluster node will update the session key to *K′_ci_*, where

(22) $$K_{\mathrm{ci}}^{\prime }=h\;(K_{\mathrm{ci}},b_{i,}N_{2})$$

Step 5: When the base station receives the packet from the cluster node, it will confirm the identity *ID_cj_* of the cluster node first. It will also search for the key *K_ci_* to decrypt *C′_ci_*

(23) $$\left(M_{f},N_{3})=D(C_{\mathrm{ci}}^{\prime },K_{\mathrm{ci}}\right)$$

The base station will then check whether *N_3_* is equal to the *N_3_* generated in step 3.

Simultaneously, the base station will update the key of the cluster node and sensor node, which will be updated to *K′_si_* and *K′_ci_*, as follows:

(24) $$K_{\mathrm{si}}^{\prime }=h(K_{\mathrm{si}},a_{i},N_{3})$$

(25) $$K_{\mathrm{ci}}^{\prime }=h(K_{\mathrm{ci}},b_{i},N_{2})$$

Step 6: After receiving the message *C_m_*, the sensor node will make use of *K′_msg_* for decryption, and will obtain the message *msg_finish_* as follows:

(26) $$\left({\mathrm{msg}}_{\mathrm{finish}},N_{1},N_{3})=D(C_{m},K_{\mathrm{msg}}^{\prime }\right)$$

The sensor node will then check whether *N_1_* is equal to the *N_1_* generated in step 1.

The previously generated keys *K_si_* and *a_i_* are used to generate a new key *K′_si_*, where

(27) $$K_{\mathrm{si}}^{\prime }=h(K_{\mathrm{si}},a_{i},N_{3})$$

*K′_si_* will be used to encrypt the transmitted data for the next transmission. When the sensor node transmits the data in the third round, the original message key *K′_msg_* will be updated to *K″_msg_*, where

(28) $$K_{\mathrm{msg}}^{\prime \prime }=h(K_{\mathrm{msg}}^{\prime },K_{\mathrm{msg}})$$

The message key *K″_msg_* and the detected message *M′_i_* are encrypted by using *K′_si_* to *C′_si_*, where

(29) $$C_{\mathrm{si}}^{\prime }=E((M_{i}^{\prime },K_{\mathrm{msg}}^{\prime \prime }),K_{\mathrm{si}}^{\prime })$$

When the sensor node transmits data for the fourth time, the message key must be updated to *K‴_msg_*, where

(30) $$K_{\mathrm{msg}}^{\prime \prime \prime }=h(K_{\mathrm{msg}}^{\prime \prime },K_{\mathrm{msg}}^{\prime })$$

The updated message key *K‴_msg_* and the detected message *M″_i_* are encrypted by using *K″_si_* to *C″_si_*,

(31) $$C_{\mathrm{si}}^{\prime \prime }=E((M_{i}^{\prime \prime },K_{\mathrm{msg}}^{\prime \prime \prime }),K_{\mathrm{si}}^{\prime \prime })$$

The session keys *K_si_*, *K′_si_* and *K″_si_* (*K″_si_* = *h*(*K′_si_*, *a*_*i*+1_, *Nounce*)) *etc*. are used for encrypting messages between the cluster node and the sensor node. In addition, the updated *K″_msg_* and *K‴_msg_* are the message keys which the cluster node uses to transmit complete messages *msg_finsh_* to the sensor node during communication.

## 4. Security and Performance Analysis

### 4.1. Security Analysis

#### 4.1.1. Security against Malicious Guessing Attacks

When a sensor network has been deployed for a certain period, the key database of the base station will be updated after a transaction so that an attacker cannot obtain the correct key to use in the next transmission. Each node includes the records of not more than three keys, which consist of two old keys and one newly generated key. When the new key is generated, the oldest key will be updated. This can improve the security of the network and reduce the memory load of the nodes.

#### 4.1.2. Security against Replay Attacks

In each communication session, including communication from the sensor node to the cluster node or communication from the cluster node to the base station, “two-way” mutual authentication is used to prevent the replay attack. We use the nonces *N*_1_, *N*_2_ and *N_3_* to check each communication message. Any communication can be determined to be legal or illegal by checking the correctness of the nonces. The related descriptions are given in steps 4.2, 5.2 and 6.2 in Figure 12. Our scheme is able to prevent replay attacks.

#### 4.1.3. Security against Falsification Attacks

To ensure secure transmission, we use the keys *K_si_* and *K_ci_* to encrypt data transmitted between the cluster node and between the cluster node and the base station, respectively. When the sensor node returns the data to the cluster node, *C*′*_si_* = *E*((*M*′*_i_*, *K*″*_msg_*, *N*_1_*′*), *K_si_*) is used for encryption. When the communication between the cluster node and the base station is finished, *K_list_* is obtained. The base station returns *K_si_* to the cluster node, and decryption begins. If the received key cannot decrypt the received packet, the received packet will be regarded as an illegal packet and will be abandoned. This practice ensures the integrity of the data transmitted, and guarantees that the data is sent from the sensor node administered by the cluster node.

#### 4.1.4. Security against Man-in-the-Middle-Attacks and Guarantee of Data Privacy

When the sensor node communicates with the cluster node, the encryption mechanism is used to prevent man-in-the-middle attacks and ensure data privacy. The transmitted message is encrypted into *C_si_* = *E*((*M_i_, K*′*_msg_*, *N*_1_), *K_si_*). The cluster node and the base station also use a similar method to prevent similar attacks and to ensure data privacy.

The attacker cannot obtain the protected data. Furthermore, the cluster node makes use of *K_msg_* to encrypt the complete message, and the message key will be updated for each transaction. Therefore, the attacker cannot imitate the cluster node to transmit a message. The man-in-the-middle-attack can thus be prevented.

#### 4.1.5. Security against Node Capture Attacks

When security is needed to transmit data between the cluster node and the sensor node or between the cluster node and the base station node, we use the keys *K_si_* and *K_ci_*, respectively, for encryption. We make use of the one-way hash function to generate the key because the one-way hash function can prevent an attacker from inverting the key. (1) *h*(*x*) is relatively easy to compute for any given *x*, making both hardware and software implementations practical. (2) For any given value *y*, it is computationally infeasible to find x such that *h*(*x*) = *y*. This is sometimes referred to in the literature as the one-way property. (3) For any given block x, it is computationally infeasible to find *z* not equal to *x* with *h*(*z*) = *h*(*x*). This is sometimes referred to as weak collision resistance. A comparison of the security and characteristics of the grid-based schemes is given in Table 2.

1. Because TTDD, CODE and DARQ do not support dynamic session-key management, they may be susceptible to various attacks.
2. In CODE and TTDD, the base stations have to reissue a query to request data or use local flooding to request data when they move out of the original grid. This will increase energy consumption and the number of collisions. DARQ and our scheme will limit the interest region to prevent a flooding storm.
3. If the base station moves out of the original grid, it reconstructs a new routing path. But CODE does not solve the routing problem when there are obstacles or voids in a sensor field.

### 4.2. Mutual Authentication

The base station uses *MAC_2_* (*h (ID_ci_*, *K_ci_) ? = MAC_2_*) to authenticate the cluster node’s identity, *MAC_3_* (*h (ID_list_*, *K_ci_) ? = MAC_3_*) to authenticate the integrity of *ID_list_* and *MAC_1_* (*h (ID_si_, K_si_) ? = MAC_1_*) to authenticate the sensor node’s identity. The reason is that the sensor node and cluster node are peer nodes. The cluster node does not store information related to the sensor node. The sensor node should therefore be authenticated by the base station. Otherwise, the cluster node can use *N_2_* to authenticate the base station. The sensor node can use *N_1_* to authenticate the cluster node. Our scheme performs mutual authentication. This makes it easier to detect an attacker.

### 4.3. Performance Analysis

Table 3 shows a comparison of the time complexity between our proposed protocol and Mizanur Rahman and El-Khatib’s scheme. Table 4 shows a comparison of the communication cost between our scheme and Mizanur Rahman and El-Khatib’s scheme. Table 5 shows a simulation which we developed based on NS2 (Network Simulation 2).

The Mizanur Rahman and El-Khatib’s scheme is based on ECC public key encryption and the random number challenge response mechanism. By contrast our scheme is based on the symmetric cryptosystem and hash function mechanism. From Table 3 and Table 4, we can see that the operation time of our scheme is faster than that of Mizanur Rahman and El-Khatib’s scheme for a large number of sensors. But the H-node end of Mizanur Rahman and El-Khatib’s scheme is superior to ours for one transaction. However, the detailed analyses are presented in our scheme. Otherwise, the Mizanur Rahman and El-Khatib’s scheme should process a key agreement procedure in advance, our scheme only uses the dynamic parameters to complete the same function without specific communication during transaction. Our scheme is simpler. Two mechanisms are different; but the differences in communication cost are not significant.

**Figure 13.**
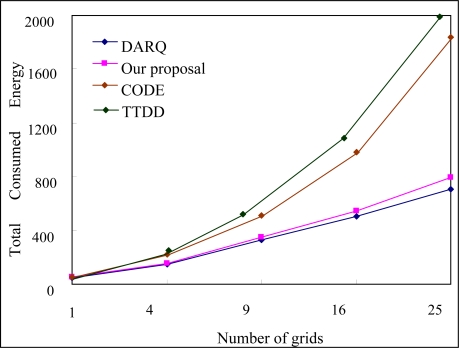
Comparison of energy consumption *versus* number of grids for different schemes.

In the following section, we compare the energy consumed in our proposed scheme to the energy consumed in other schemes. A comparison of the total energy consumption in other schemes for various different numbers of nodes is shown in Figure 13. As can be seen, the total energy consumed in the proposed scheme and in the DARQ, CODE and TTDD schemes increased when the number of grids increased. However, the total energy consumed in our scheme is less than the energy consumed in the CODE and TTDD schemes, but is more than the DARQ scheme. Since our scheme uses a grid-based mechanism to restrict the possibility of packet flooding, such a result meets our expectations. However, although our scheme is based on the DARQ scheme, more energy is consumed in encryption computation.

## 5. Conclusions

We proposed an efficient management mechanism for WSNs that includes the following benefits:

1. The proposed mechanism can significantly conserves the memory of a sensor node.
2. Dynamic key management for each data transmission is used only once. This method reduces the probability of an attacker guessing a key correctly. The method thus improves security.
3. The total energy consumed in our scheme is less than the energy consumed in the other schemes except the DARQ scheme.
4. The proposed scheme uses a grid-based approach. In addition to using grids, we also limited the flooding region to decrease the overhead for routing discovery in order to reduce the probability of a flooding storm.

In future research, we will propose a solution to find a routing detour around void regions. Void regions exist in a network because some grids do not deploy sensors. The design of multiple interest regions will be taken into consideration to provide data aggregation for WSNs.

## Figures and Tables

**Table 1.** Equivalent key sizes for symmetric and asymmetric cryptography.

**Bits of Security**	**Symmetric Algorithm**	**RSA**	**ECC**
80	2TDEA	k = 1,024	f = 160–223
112	3TDEA	k = 2,048	f = 224–255
128	AES-128	k = 3,072	f = 256–383
192	AES-192	k = 7,680	f = 384–511
256	AES-256	k = 15,360	f = 512+

**Table 2.** The security and characteristic comparison of the grid-based schemes.

**Grid-based scheme**	**TTDD [15,16]**	**CODE [17]**	**DARQ [20,21]**	**Our proposal**
**Attacks**				
Against malicious guessing attacks	NA	NA	NA	Yes
Against replay attacks	NA	NA	NA	Yes
Against falsification attacks	NA	NA	NA	Yes
Against man-in-the-middle-attacks and guarantee of data privacy	NA	NA	NA	Yes
Against node capture attacks	NA	NA	NA	Yes
Grid-based protocol	Yes	Yes	Yes	Yes
Event-driven data dissemination	Yes	Yes	Yes	Yes
Limit interest region to prevent flooding storm	No	No	Yes	Yes
Routing problem with obstacles in sensor field	Yes	No	Yes	Yes
Routing problem with voids in sensor field	Yes	No	Yes	Yes

**Table 3.** Comparison of time complexity between our scheme and Mizanur Rahman and El-Khatib’s scheme.

Nodes Relationship	Mizanur Rahman and El-Khatib’s scheme [23]	Our scheme
Sensor node (or L-node)	*T_KEY_+ 2T_h_ + T_V_ + T_EECC_*	*1T_E_ + 1T_D_ + 5T_h_*
Cluster node (or H-node)	*T_KEY_ + T_EECC_+ 2T_h_ + T_V_*	*3T_E_+2T_D_ + 4T_h_ + 1T_DIV_+tT_ADD_*
Base station	N/A	*1T_E_ +2T_D_+ 3T_V_+ 5T_h_*

Notes:
*T_E_*: the time complexity for using a symmetric encryption algorithm *T_D_*: the time complexity for using a symmetric decryption algorithm *T_V_*: the time complexity for verifying a message *T_h_*: the time complexity for using a hash function *T_ADD_*: the time complexity for addition *T_DIV_*: the time complexity for division *t*: the number of aggregation data for a cluster node *T_KEY_*: the time complexity for generating an ECC secret key *T_EECC_*: the time complexity for ECC encryption

**Table 4.** Comparison of communication cost between our scheme and Mizanur Rahman and El-Khatib’s scheme.

Nodes Relationship	Mizanur Rahman and El-Khatib’s scheme[23]	Our scheme
Sensor node and Cluster node (or H-node)	*2T′_R_ +2T′_h_+ 2T′_EECC_*	*2T′_E_ + 1T′_MSG_+ 1T′_h_*
Cluster node (or H-node) and Base station	N/A	*3T′_E_ + 2T′_MSG_+ 3T′_h_*
H-node and H-node	*T′_EECC_ +4T′_R_+2T′_h_*	N/A

Notes:
*T′_E_*: the transmission time for a symmetric encryption message (For example AES-128 bits) *T′_MSG_*: the transmission time for a message (16 bits, for example *ID_si_, ID_ci_*) *T′_h_*: the transmission time for a hash message (for example, hash message of 168 bits) *T′_EECC_*: the transmission time for an ECC ciphertext (for example, a ciphertext of 256 bits) *T′_R_*: the transmission time for a random number (for example, random number of 16 bits)

**Table 5.** Parameters used in the simulation environment.

**Parameter**	**Values**
Simulation tool	NS2
Simulation area	2,000 m × 2,000 m
Number of nodes	100–400 nodes
Base station mobility model	Random waypoint model
Radio transmission range	100 m
Data packet size	64 bytes
Data transmission rate	1 Mbps
